# Assessing Bioprinted Functionalized Grafts for Biological Tendon Augmentation In Vitro

**DOI:** 10.3390/ijms25094752

**Published:** 2024-04-26

**Authors:** Cristina Del Amo, Miguel Perez-Garrastachu, Ines Jauregui, Xabier Llama-Pino, Isabel Andia

**Affiliations:** 1 Regenerative Therapies, Biobizkaia Health Research Institute, 48903 Barakaldo, Bizkaia, Spain; cristina.delamomateos@bio-bizkaia.eus (C.D.A.); miguel.perez@ehu.eus (M.P.-G.); x.llamapino@gmail.com (X.L.-P.); 2 3D Printing and Bioprinting Lab, Biobizkaia Health Research Institute, 48903 Barakaldo, Bizkaia, Spain; ines.jaureguimonasterio@bio-bizkaia.eus; 3 Department of Cell Biology and Histology, Faculty of Medicine and Nursing, UPV/EHU, 48940 Leioa, Biscay, Spain

**Keywords:** extrusion bioprinting, tendon, grafts, platelet-rich plasma, inflammation, angiogenesis

## Abstract

Tendinopathy, characterized by inflammatory and degenerative changes, presents challenges in sports and medicine. In addressing the limitations of conservative management, this study focuses on developing tendon grafts using extrusion bioprinting with platelet-rich plasma (PRP)-infused hydrogels loaded with tendon cells. The objective is to understand paracrine interactions initiated by bioprinted tendon grafts in either inflamed or non-inflamed host tissues. PRP was utilized to functionalize methacrylate gelatin (GelMA), incorporating tendon cells for graft bioprinting. Bioinformatic analyses of overexpressed proteins, predictive of functional enrichment, revealed insights into PRP graft behavior in both non-inflamed and inflamed environments. PRP grafts activated inflammatory pathways, including Interleukin 17 (IL-17), neuroinflammation, Interleukin 33 (IL-33), and chemokine signaling. Interleukin 1 beta (IL-1b) in the graft environment triggered p38 mitogen-activated protein kinase (MAPK) signaling, nuclear factor kappa light chain enhancer of activated B cells (NF-kB) canonical pathway, and Vascular Endothelial Growth Factor (VEGF) signaling. Biological enrichment attributed to PRP grafts included cell chemotaxis, collagen turnover, cell migration, and angiogenesis. Acellular PRP grafts differed from nude grafts in promoting vessel length, vessel area, and junction density. Angiogenesis in cellular grafts was enhanced with newly synthesized Interleukin 8 (IL-8) in cooperation with IL-1b. In conclusion, paracrine signaling from PRP grafts, mediated by chemokine activities, influences cell migration, inflammation, and angiogenic status in host tissues. Under inflammatory conditions, newly synthesized IL-8 regulates vascularization in collaboration with PRP.

## 1. Introduction

Conditions affecting tendons present a noteworthy aspect of activity-related disorders, presenting considerable clinical challenges in both sports and modern medicine [1]. Within this spectrum, tendinopathy encompasses a wide array of inflammatory and degenerative changes, including partial and full-thickness tears, which are typically accompanied by pain and a gradual decline in functional capacity [2]. In the initial phases of tendinopathy, conservative management strategies primarily involve activity modification and exercise [3]. However, as tendinopathy progresses to a point where conventional treatments prove inadequate to halt tendon degeneration, alternative approaches, including platelet rich plasma (PRP) applications [4,5] or biological augmentation with grafts, are sought [6]. Extrusion bioprinting enables the additive manufacturing of personalized cellular tendon grafts, offering a means to repair tendon tears and foster healing. Furthermore, in vitro studies of these grafts contribute to understanding the potential of tenocytes in tendon repair and the biological processes they can activate through paracrine activities upon implantation [7]. These grafts serve as valuable tools for enhancing our understanding of tenocyte roles in pathological processes, both in the presence and absence of inflammation.

The existence of inflammation in chronic tendon disorders remains a topic of ongoing debate [8]. Recent systematic reviews exploring inflammation’s role in tendinopathy pathogenesis have revealed a range of conditions, including chronic inflammation and non-inflammatory degeneration [8]. While various inflammatory biomarkers were identified in two-thirds of the reviewed articles, a common pathophysiology remained elusive. Inflammation is intricate, characterized by two primary types that represent opposite ends of the spectrum. Type I denotes harmful inflammation driven by cytokines such as Tumor Necrosis Factor (TNF-a), Interferon gamma (IFN-gamma), and Interleukin (IL) 1 beta (IL-1b). Conversely, type II, indicative of reparative inflammation, is associated with IL-5, IL-33, IL-4, and IL-10. Additional cytokines, mainly IL-6 and IL-8, are pleiotropic with different functions depending on the specific molecular context.

Not only immune cell infiltration patterns but local tendon cells play a role in shaping the cytokine microenvironment [9]. Research unveils that tenocytes isolated from human supraspinatus change the expression level of surface markers (Intercellular Adhesion Molecule (ICAM-1), Vascular Cell Adhesion Molecule (VCAM-1), Major Histocompatility Complex (HLA-ABC, and HLA-DR) and their cytokine release (IL-6, IL-8, and Monocyte Chemotactic Protein (MCP-1)) profile in response to activated human immune cells [9]. Other authors [10] have demonstrated that in response to inflammation, tenocytes acquire a pro-inflammatory phenotype. Similarly, new data indicate that mesenchymal stromal cell polarization (MSC1 and MSC2) is mediated by specific Toll Like Receptor (TLR) stimulation [11].

Understanding the interplay and timing of type I and II inflammatory programs is essential for controlling tendon repair adequately, as overactivation of either can lead to tendinopathy [2,12]. Maintaining a balance between type I and II inflammation is crucial for effective healing [13]. In tendinopathies, an imbalance favoring type I inflammation, exacerbated by IL-1b, may worsen tissue properties by inducing leukocyte infiltration and matrix metalloproteinases, leading to extracellular matrix degradation and tear development [14]. 

This study aims to develop a biomimetic tendon graft using 3D bioprinting with PRP-infused bioinks loaded with tendon cells. For in vitro pre-clinical studies, we focused on disc-like scaffold geometry with specified dimensions. To comprehend the paracrine interactions initiated by the graft composed of tendon cells embedded in Gelatin methacryloyl (GelMA) infused with PRP, in the host tendon, we examined the potential inflammatory response post-implantation in a non-inflamed environment in vitro. Subsequently, to simulate an inflammatory host tissue environment, the PRP grafts were exposed to IL-1b. In both scenarios, we scrutinized the detailed molecular responses using protein arrays and bioinformatics. Furthermore, laboratory experiments were conducted for additional validation.

## 2. Results

### 2.1. Tendon Stromal Cells Adhesion and Viability within PRP Grafts

**Figure 1:**
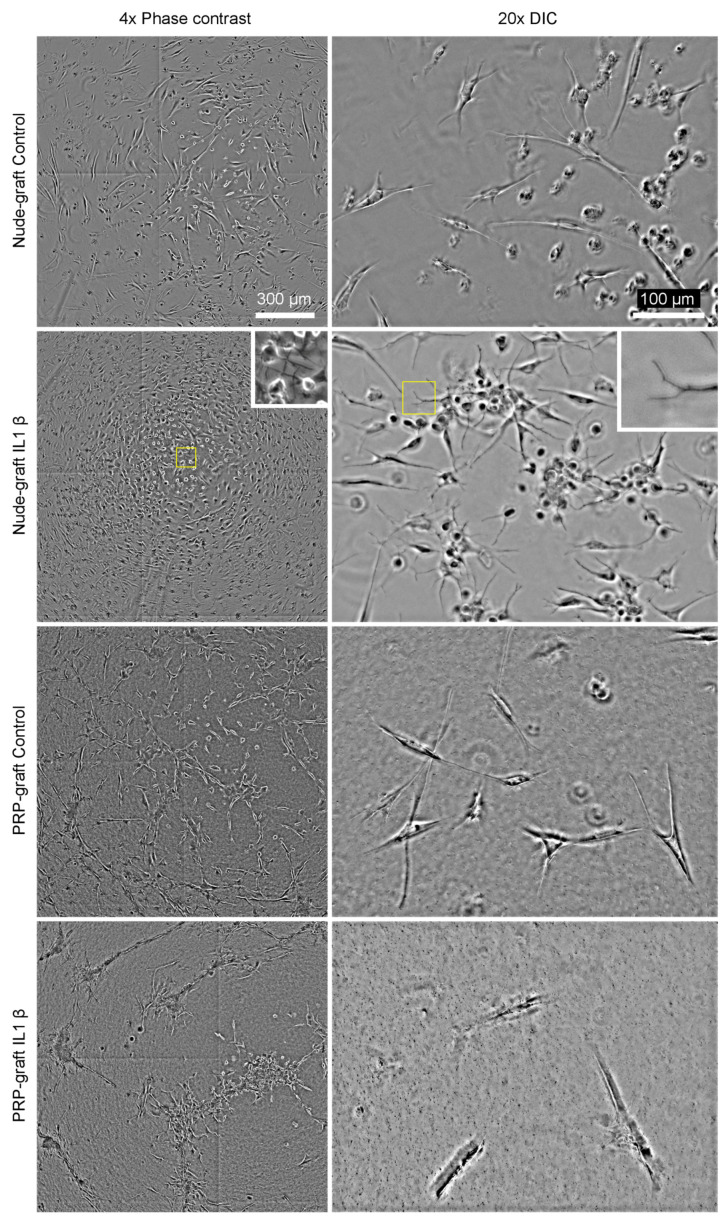
Micrographs of tenocytes cultured over nude or PRP-supernatant doped constructs were taken using phase contrast at 4× and differential interference contrast (DIC) at 20×. The cultures were maintained under normal conditions (control) or in an inflamed (IL-1β) environment for 96 h. Insets in the second row magnify the region of interest highlighted in yellow to emphasize dendritic structures resulting from inflammation. The scale bar is 300 µm for the photomicrographs in the left column and 100 µm for those in the right column.

The initial tendon stromal cell (moving forward, we will refer to these cells as tenocytes) culture experiments were based on qualitative observations of growth patterns when cultured on the graft. The experiments revealed that tenocytes grow normally on either matrix, but subtle differences are apparent (see Figure 1). Specifically, tenocytes tend to grow uniformly on the nude matrix, exhibiting their characteristic spindle-shaped morphology and radiating extensions on the bare matrix (20 × DIC nude graft control, Figure 1). Tenocytes behave similarly when grown on a PRP-doped matrix, but we can subtly note that they tend to cluster in nodes (20 × DIC PRP graft control, Figure 1). However, under inflammatory conditions, they exhibit significant differences. When grown on a nude matrix, they extend numerous dendrite-like prolongations and increase their overall surface area (inset nude graft IL-1b). Interestingly, the opposite occurs when they grow on PRP graft. They tend to cluster and establish junctions between nodes but leave large areas of matrix without cells or sparsely populated.

#### Viability of Tendon Cells within Bioprinted Grafts

**Figure 2:**
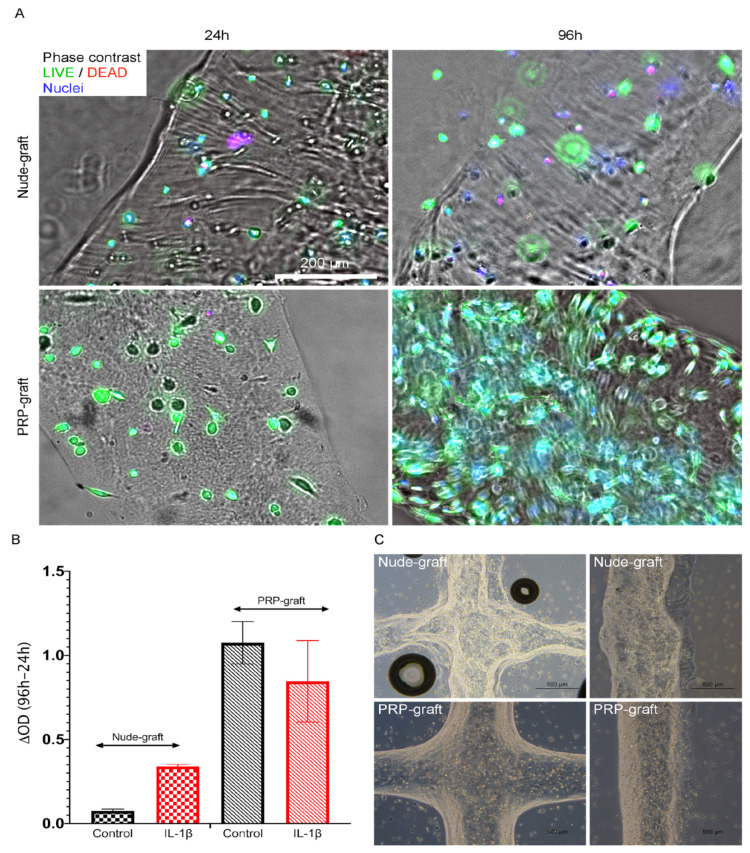
Assessment of cell viability post-bioprinting: extrusion through a 20 G conical tip at 30–50 kPa pressure and 10 °C bed temperature, followed by 405 nm photocuring. The introduction of 50% PRP into GelMA improves cell viability, as demonstrated by photomicrographs (**A**) and XTT assay results (**B**). (**C**) provides further insight into the infill pattern. Both GelMA-PRP and GelMA (nude) grafts were loaded with an equal cell number (500,000 cells/mL) (**A**,**C**). A total of 30 uL of each bioink was used in all XTT experiments, ensuring that both PRP and nude conditions were loaded with an equal cell number (15,000 cells/well) (**B**). The PRP condition showed increased cell proliferation, as indicated by the metabolic activity observed in the XTT assay and depicted in (**B**), inflammatory conditions are depicted in red color.

Tendon cells in PRP grafts showed enhanced viability compared to those in nude grafts, as indicated by the XTT assay (Figure 2A,B). IL-1b exposure did not negatively impact tenocyte viability. IPA^®^ analyses predicted increased cell survival (Z = 3.315, *p* = 3.58 × 10^−71^) and viability (Z = 3.368, *p* = 7.5 × 10^−70^), with reduced apoptosis (Z = −2.196, *p* = 5.37 × 10^−81^) in PRP grafts. Additionally, the analyses predicted similar cell survival (Z = 2.185, *p* = 3.58 × 10^−71^) and viability (Z = 2.469, *p* = 7.5 × 10^−70^) under inflammatory conditions (IL-1b exposure). In all observations, reflecting the avascular nature of tendons, the IPA knowledgebase anticipated the activation of hypoxia signaling (Hypoxia Inducible Factor alpha, HIF-1) with Z-scores of 4.082 for PRP grafts and 3.266 inflamed PRP grafts. Under these circumstances, tendon cells generated Vascular Endothelial Growth Factor (347 pg/mL, VEGF-A), a crucial angiogenic factor. Upon infusing grafts with PRP, VEGF levels rose with the platelet dosage but stayed below those in nude grafts (167 pg/mL and 253 pg/mL extruded from PRP grafts containing 30 and 200 platelets per bioink mL, respectively). 

### 2.2. Predicted Protein–Protein Interactions and Networks for PRP-Infused Grafts: STRING Database

Protein interaction enrichment analysis was performed on data obtained from the ELISA arrays. We compared the enrichment of pathways in GelMA PRP-infused grafts to GelMA grafts (nude). The connectome of PRP grafts, comprising 122 nodes and 113 edges, exhibited an average node degree of 1.85, with a protein–protein interaction (PPI)-enrichment *p*-value < 1.0 × 10^−16^.

**Figure 3:**
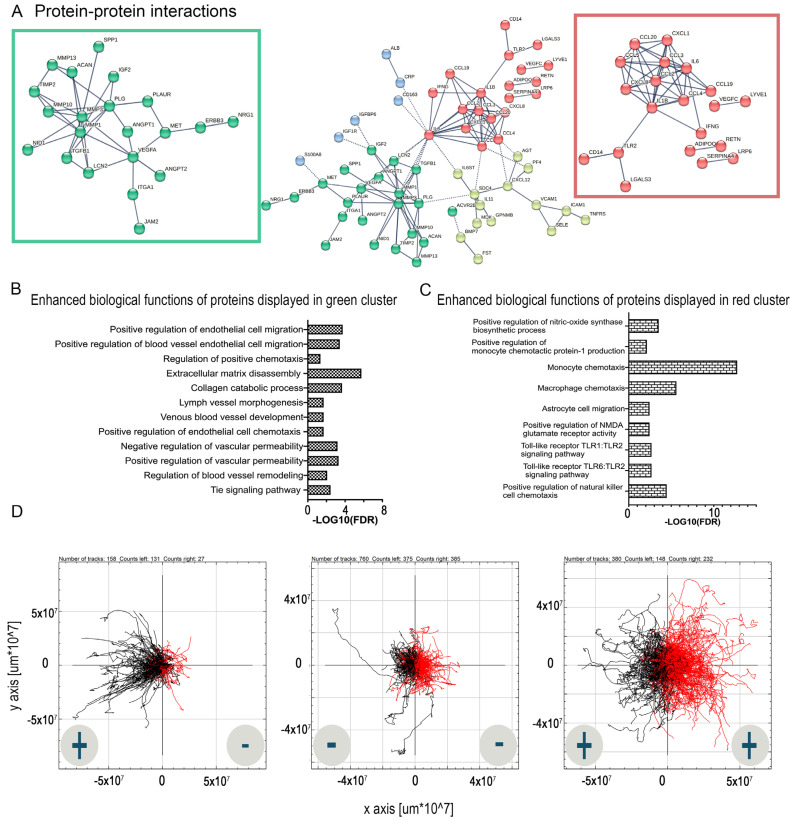
(**A**) shows STRING interaction network for the proteins released from PRP grafts. The four clusters that were identified using k means are shown in colors. Angiogenic protein−protein interactions (**A**) (in green) and corresponding functional enrichment (**B**); chemokine interactions (**A**) (in red) and associated functional enrichment (**C**); (**A**) cluster in blue is involved in acute inflammatory response and cluster in yellow in CXCR chemokine receptor binding. (**D**) shows HUVEC migration toward PRP stimuli. The +/− symbols represent the presence/absence of the chemoattractant proteins.

Due to their potential functional importance post-implantation, we specifically focused on two significant clusters (highlighted in red and green in Figure 3A). The angiogenesis or green cluster, with 30 nodes and 38 connections, had an average node degree of 2.53 with a PPI-enrichment *p*-value < 1.0 × 10^−16^. Functional enrichment in this network suggests processes related to angiogenesis. Predictions of positive regulation of endothelial cell migration and the regulation of positive chemotaxis are attributed to the PRP component of the graft. In vitro confirmation tests showed significant HUVEC migration towards PRP, supported by a Rayleigh test, *p* < 0.05, and a forward migration index higher than (+/+) and (−/−) controls (Figure 3D).

The chemokine or red cluster comprised 35 proteins with 45 connections, an average node degree of 2.57, and a PPI enrichment *p*-value < 1.0 × 10^−16^ (Figure 3B,C). Enriched biological functions relevant to tissue healing include monocyte and macrophage chemotaxis and Toll-like Receptor (TLR) signaling pathways.

### 2.3. Analyses of PRP Grafts in the IPA^®^ Knowledgebase

To evaluate the influence of PRP in PRP grafts or inflamed PRP grafts, comparisons were made with nude grafts or inflamed nude grafts, respectively. 

#### 2.3.1. Activated Canonical Pathways in the IPA^®^ Knowledgebase

**Figure 4:**
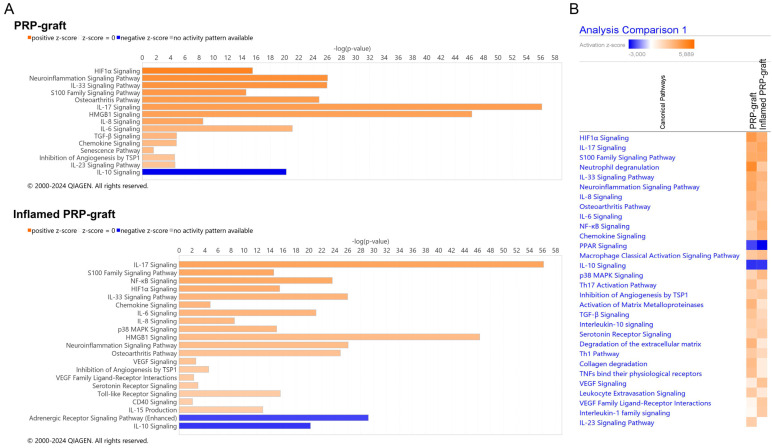
**A** illustrates canonical pathways represented according to Z-scores ranging from 2 to 4.9 for PRP grafts (**A**) and from 2 to 5.89 for inflamed PRP grafts (absolute values) with significant levels depicted on the *y*-axis. (**B**) Analysis comparison between PRP grafts and inflamed PRP grafts. Images generated by QIAGEN IPA (QIAGEN Inc., https://digitalinsights.qiagen.com/IPA, accessed on 22 April 2024) IPA software.

In the overall molecular network extruded from the PRP grafts, the top canonical pathways are shown in Figure 4A. PRP grafts can activate, through paracrine mechanisms, various relevant inflammatory pathways, including IL-17, neuroinflammation, IL-33, IL-8, and IL-6. At the same time, anti-inflammatory IL-10 signaling is downregulated (Table 1). Corroborating STRING predictions, the IPA^®^ knowledgebase predicted the activation of immune mechanisms, mainly neutrophil and monocyte/macrophage infiltration.

#### 2.3.2. Response of PRP Grafts in Inflamed Environments

To comprehend the behavior of PRP grafts in the presence of inflammation, a heat-map analysis was conducted. Inflamed PRP grafts showed increased interactions in the VEGF family ligands (from Z = 0.44 to Z = 2.23) and VEGF signaling (from Z = 0.818 to Z = 2.45), with no alterations in the inhibition of angiogenesis by Thrombospondin (TSP-1) (Z = 2.0 and 2.236). Concurrently, the NF-kB pathway, p38-MAPK (Z = 1.976 to 3.413 and Z = 1.807 to 2.840, respectively), and CD40 signaling were also enhanced (Z = 1.00 to 2.00), while TGF-b1 signaling decreased (Z = 2.449 to 1.633) (Figure 4B). In IPA^®^, the significance of pathways is determined by the *p*-value of overlap, computed through the right-tailed Fisher’s Exact Test. This significance reflects the likelihood of molecules from graft-conditioned media being associated with the canonical pathway solely by random chance.

### 2.4. Impact of IL-1b Exposure on PRP Grafts: Meaningful Activated Pathways in the Context of Tendinopathy 

**Figure 5:**
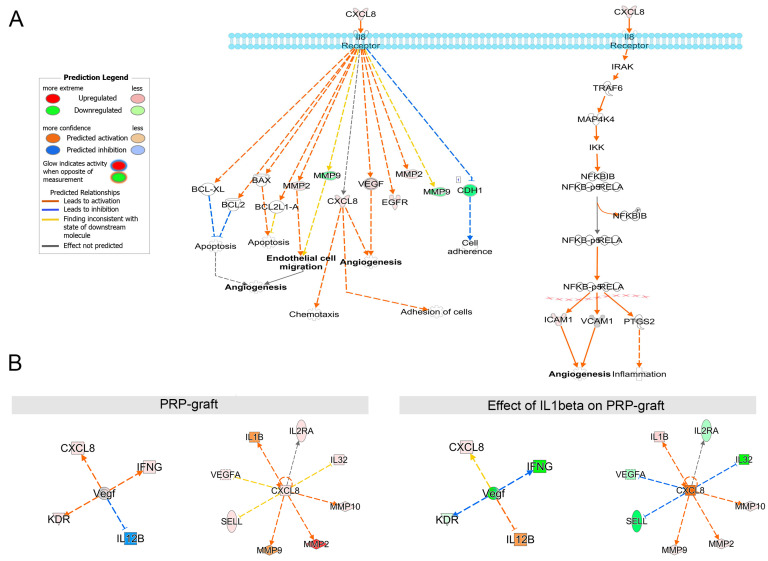
IL-8 pathways describing the reactions in the cell that lead to angiogenesis, cell migration, and inflammation; (**A**) IL-8 (CXCL8) has a regulatory role in angiogenesis and inflammation; (**B**) proteins do not act as independent entities. The figure shows interactions based on literature mining. Images generated by QIAGEN IPA (QIAGEN Inc., https://digitalinsights.qiagen.com/IPA, accessed on 22 April 2024) IPA^®^ software.

By utilizing the IPA knowledgebase, we investigated the molecular mechanisms triggered by IL-1b exposure, uncovering an overexpression of CXCL8, Interleukin 8 (IL-8). IL-8 promotes the upregulation of MMPs (-2, -9, and -10) in both pink (assessed) and orange (predicted) categories while simultaneously inhibiting VEGF, SELL, and IL-32 (Figure 5).

#### Matrigel Assay

**Figure 6:**
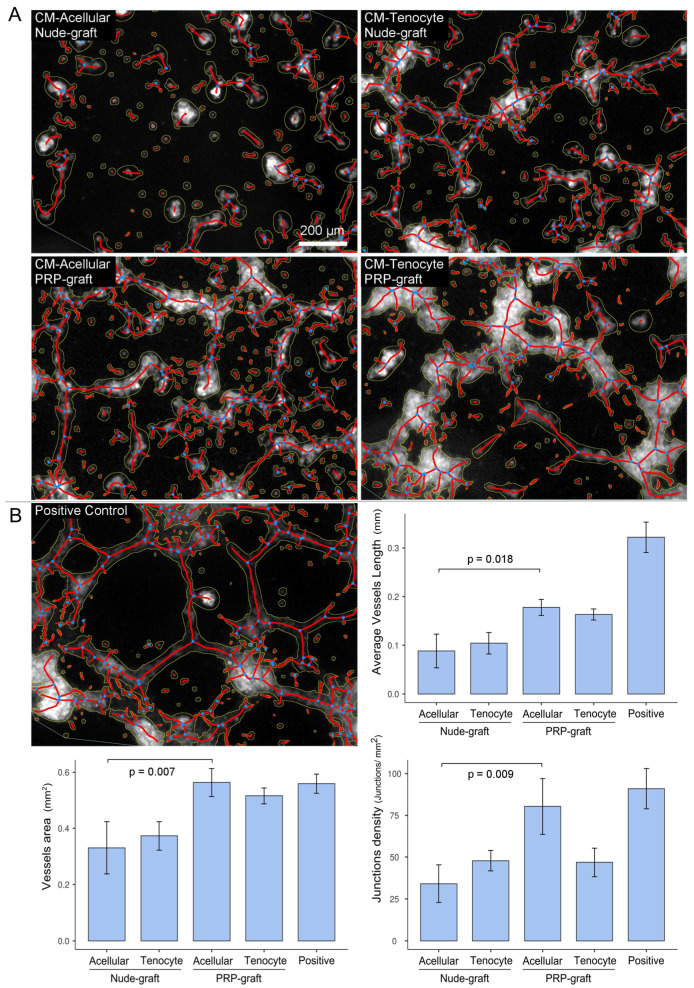
Assessment of the angiogenic potential of HUVEC after exposure to conditioned media collected under inflammatory conditions (IL-1b 50 ng/mL). (**A**) Detected skeletons (in red) of HUVEC exposed to inflammatory conditioned media (CM) from acellular nude grafts and PRP grafts or cellular nude grafts and PRP grafts. (**B**) On the left is a representative field of HUVECs grown over a matrigel layer cultured with complete growth medium (positive control). Bar graphs illustrating the mean vessel length (mm), vessel area (mm^2^), and junction density (junction/mm^2^) for each experimental condition: acellular nude grafts, acellular PRP grafts, cellular nude grafts, cellular PRP grafts, and positive control (Pos). Blue dots represent junctions.

We employed matrigel tube formation assays as optimized screening methods to investigate whether media conditioned from both GelMA (nude) or GelMA-PRP grafts facilitated or hindered angiogenesis (Figure 6).

Due to the elevated IL-8 concentrations in the conditioned media of cellular grafts, we investigated the conditioned media of acellular grafts exposed to IL-1β, encompassing both nude and PRP grafts. Notably, the latter exhibited distinctions from nude grafts, displaying a notable increase in the promotion of vessel length (*p* = 0.018), vessel area (*p* = 0.007), and junction density (*p* = 0.009). These results emphasize the pronounced angiogenetic stimulatory effect of PRP in comparison to the hypothesized angiogenic impact of IL-1β.

However, conditioned media from inflamed cellular grafts, characterized by high IL-8 concentration, whether PRP-infused or nude, did not reveal significant differences in vessel area (*p* = 0.083), vessel length (*p* = 0.106), and junction density (*p* = 0.954). It is noteworthy that acellular or cellular PRP grafts exhibited no variances in vessel area compared to the positive control, and junction density remained consistent between acellular PRP grafts and the positive control. When contrasting acellular and cellular PRP grafts, the latter, marked by higher IL-8 levels, exhibited a reduced number of junctions (*p* = 0.050).

## 3. Discussion

Tendons, due to their low cellularity and avascular nature, have limited healing resources and require enhancement by augmentation strategies. These strategies can include structural, biological, or mixed augmentation methods to improve tendon healing. In this study, we chose to manufacture PRP grafts for biological augmentation with a focus on regulated reparative inflammation [15]. To achieve this, we formulated a bioink based on gelatin methacryloyl (GelMA) combined with PRP and loaded with tendon cells. This allowed us to bioprint biologically enriched regenerative grafts for tendon repair.

Gelatin, a denatured form of collagen, lacks mechanical strength due to the loss of its triple helix structure. However, its mechanical properties can be adjusted through chemical crosslinking with methacrylic anhydride (MA) and polymerization using a photocuring agent (LAP) and UV light [16]. Researchers have utilized GelMA for tendon conditions. For example, a study by Han Q et al. in 2024 [17] used GelMA microspheres loaded with a heparin–dopamine conjugate and Hepatocyte Growth Factor (HGF) to mitigate oxidative stress and inflammation in tendinopathy. Similarly, Donderwinkel I in 2023 [18] evaluated decellularized ECM combined with GelMA, encapsulated Bone Marrow Stromal Cells (BMSCs), and supplemented with Transforming Growth Factor Beta 3 (TGF-b3) to induce tenogenic differentiation under mechanical stimulation.

Grafts utilized for biological augmentation are often paired with interventional procedures like bone marrow stimulating techniques. For example, GelMA loaded with kartogenin has been combined with bone marrow stimulating techniques, such as microfractures, to stimulate fibrocartilage formation at the enthesis in treating rotator cuff conditions [19]. Kartogenin promotes cartilage-like tissue and has been used in conjunction with PRP in treating injured tendon entheses [20]. PRP contains numerous signaling factors that can enhance regenerative processes in tendon conditions. In our study, the encapsulated tenocytes responded to the PRP signaling pool by synthesizing additional cytokines, chemokines, and growth factors, including RANTES, IL-8, IL-6, MCP-1, VEGF, and HGF [7]. Hence, these grafts provide the pathological tissue with tendon cells that participate in healing mechanisms through paracrine actions. Moreover, GelMA, often used as a cell carrier [21], also serves as a vehicle for the delivery of additional PRP molecules.

Our PRP grafts are designed to restore tendon homeostasis by inducing reparative inflammation and angiogenesis. Clinical experience suggests that intratendinous PRP injections exacerbate inflammation in the short term, which we hypothesize is a necessary step in restoring homeostasis, reducing pain, and improving function [5,22,23,24]. However, there is a lack of knowledge about the molecular mechanisms that define reparative inflammation.

Tenocytes synthesize various inflammatory cytokines in response to injury, but it is controversial whether specific types of inflammation are necessary for tendon healing and tendinopathy homeostasis recovery. Tendinopathy is often considered a failed healing response due to the tissue’s inability to terminate inflammation [5].

We hypothesize that the activation of both IL-17 and IL-33 could be a potential mechanism of action of PRP to modulate inflammation in tendons. IL-33 has been proposed as a significant cytokine in the transition from type-1 to type-3 collagen synthesis and in ECM remodeling [25,26]. IL-33 deficiency leads to persistent inflammation [27]. Conversely, IL-17 and its receptors are present in both healthy and tendinopathic tenocytes, and IL-17A enhances the activation of p38 and NF-kB in tendon fibroblasts [28].

The downstream effects could include the activation of additional homeostatic inflammatory pathways, such as IL-6 and IL-8 [29]. Furthermore, IL-17 induces the downstream synthesis of MMP-3 in tendon cells [28]. Notably, we have found that tendon cells within PRP grafts maintain viability regardless of the activated inflammatory pathways.

Activated tenocytes upregulate the secretion of inflammatory cytokines and chemokines involved in T-cell recruitment and activation, including IL-6, IL-8, MCP-1, and RANTES [30]. It is hypothesized that the cross-talk between activated T cells and tenocytes creates a self-amplifying loop [30].

Dysregulated immunobiology is implicated in conditions such as the frozen shoulder [31] with a switch from macrophages to predominance of IL-17A-producing T cells. In fact, IL-17 family members are proposed as amplifiers of tendon inflammation [28], and IL-17A inhibition has been suggested for frozen shoulder. 

However, despite the common perception of type I and type II inflammation as dichotomous, current research in T-cell polarization indicates the need to reposition our understanding of type 1, 2, and 3 inflammatory responses [32]. This paradigm enables immune responses to adapt to changing circumstances based on different stimulating or inhibitory cytokines. Further research is needed to capture complex situations in which a given cytokine, such as IL23 (also known as IL17E), is involved in both type 1 and 3 responses [32]. 

In addition to regulating inflammation, PRP grafts also play a significant role in angiogenesis, which is crucial for tissue repair. This multistep process involves the degradation of the extracellular matrix (ECM), the migration of endothelial cells, the organization of microtubules, and the sprouting of new capillary branches [33].

Our data indicate that PRP grafts can positively influence angiogenesis. They do this by promoting the migration of endothelial cells and enhancing the area and junction density of vessels. A key player in this process is VEGF, which is a master regulator of angiogenesis. VEGF stimulates the proliferation and migration of endothelial cells and directly controls angiogenesis. Simultaneously, the activation of Matrix Metalloproteinase-2 (MMP-2) indirectly supports vessel growth [34].

Another indirect factor in angiogenesis is IL-8, which is triggered by the presence of IL-1β in the microenvironment. IL-8 can potentially activate VEGF and the Matrix Metalloproteinases MMP-2 and MMP-9, thus promoting angiogenesis through intermediary mechanisms.

Interleukin-1 beta (IL-1b) is a well-studied cytokine with a long-established role in managing both angiogenesis and inflammation. The dual functionality of IL-1b is critical in physiological contexts where angiogenesis and inflammation concurrently occur, such as during tissue repair and regeneration. IL-1b orchestrates these processes by regulating multiple cellular pathways, thereby influencing the behavior of different cell types involved in these phenomena.

One particularly significant facet of IL-1b’s regulatory role lies in its interaction with VEGF, a master regulator of angiogenesis. Both IL-1b and PRP are known to regulate shared cellular pathways, a notable one being the NF-kB cascade [35]. The concurrent induction of the NF-kB cascade by IL-1b and PRP creates an environment conducive to both inflammation and angiogenesis [36]. This signifies a potential mechanism by which IL-1b and PRP can collectively regulate these processes, resulting in the overlapping effects observed in inflammatory and angiogenic signaling.

Thus, the interplay between IL-1b and PRP, particularly through the NF-kB pathway, provides a possible explanation for the simultaneous regulation of angiogenesis and inflammation. This finding underscores the complexity of these processes and the intricate balance required for successful tissue repair and homeostasis.

There are a multitude of tendon and enthesis conditions that could potentially benefit from the application of PRP grafts. These applications could be performed either through surgical means or via minimally invasive procedures. The most evident and widely recognized application is perhaps the augmentation of rotator cuff repair. However, other tendons, such as the Achilles and gluteal tendons, could also be viable candidates for PRP graft augmentation. Nevertheless, it is important to note that structural augmentation often takes precedence over biological augmentation. That being said, a recent study has shed light on the significance of concurrent biological augmentation. The study showed that commercial extracellular matrix patches like Permacol^®^, which is a porcine dermis graft, and GraphtJacket^®^, a cadaveric human dermis, demonstrated extracellular matrix disruption at four weeks post-implantation. Interestingly, these commercial patches did not show any clear advantages over the control group, as per the study conducted by [37]. These point to the necessity of further research and exploration in the field.

A limitation of our study is the focus on early inflammation without measuring collagen synthesis from tendon cells embedded within the grafts. The proof of concept entails implanting grafts into Achilles tendons using a humanized murine model. Conducting histological analyses at different time points would deepen our understanding of the graft’s angiogenic effects, the host tissue’s inflammatory response [38], GelMA remodeling, and ECM generation.

Nevertheless, further research is needed to fully understand the role of PRP grafts in tendon repair and to optimize their application in various clinical settings. This includes exploring different methods of application, such as minimally invasive procedures, and investigating their effectiveness in treating different types of tendon and enthesis conditions.

Biomechanical analyses were not considered since the graft is specifically designed to address biological augmentation of tendon repair rather than structural augmentation.

## 4. Methods and Materials

### 4.1. Primary Culture of Human Tenocytes

Tissue fragments were gathered from the semitendinosus tendon during the surgical reconstruction of the anterior cruciate ligament (ACL) after obtaining informed consent and approval from the local ethics committee. Tendon cells were isolated using an explant procedure as described previously [7]. 

### 4.2. Preparation of PRP 

Citrate phosphate dextrose (CPD) anticoagulated whole blood was collected by apheresis from five healthy donors and processed by the Basque Biobank (Centro Vasco de Transfusión, Galdakao, Bizkaia, Spain; CEIC n° CES-BIOEF 201907). Leuko-reduced platelet-rich plasma (PRP) and platelet-poor plasma (PPP) were generated using the REVEOS automated blood processing system from Terumo BCT Inc. (Lakewood, CO, USA). PRP samples from the blood bank were diluted 1:5 in PPP. Supernatants were prepared by adding 10% CaCl_2_ to achieve a final concentration of 22.6 mM, followed by incubation at 37 °C for one hour, as previously described [39]. Subsequently, the mixture was centrifuged, and the supernatant was filtered through 40 µm filters, aliquoted, and stored at −80 °C.

### 4.3. Preparation of Functionalized GelMA

The GelMa stock solution was formulated by combining sterile lyophilized GelMa (CellInk PhotoGel^®^ 50% FD, headquarters) with sterile filtered DMEM-F12, supplemented with 0.5% (*w*/*v*) LAP (Lithium phenyl-2,4,6- Trimethylbenzoylphosphinate), resulting in a final concentration of 10%. The solution was allowed to rest at 40 °C overnight with gentle agitation and protection from light until fully dissolved, ensuring no trapped air bubbles. Following complete dissolution, the GelMa stock solution was stored in the dark at 4 °C and utilized within one month.

For the preparation of bioinks, the GelMa stock was heated in a culture oven at 37 °C for a minimum of 30 min until liquefied. Subsequently, it was diluted to a final concentration of 5% by adding an equal volume of either preheated DMEM-F12 or the extruded supernatant from PRP clots, all maintained at 37 °C.

### 4.4. Bioprinting of Tendon Grafts

Tissue grafts were manufactured employing a CellInk BioX bioprinter. To prepare the bioink, GelMa stock at 10% concentration and the corresponding supplement (DMEM of PRP-derived secretome) were mixed with a cell concentrate (3,000,000 tenocytes/mL) in a ratio of 50:40:10. It is crucial to warm all components to 37 °C before the mixing process. After preparation, the bioinks were loaded into Cellink 3 mL pneumatic syringes and briefly stored in a freezer for two minutes to expedite gelling and prevent cell precipitation. Following gelatinization, the syringes were placed in a thermostated bioprinter head at 22 °C for a minimum of 5 min to ensure even temperature distribution throughout the cartridge.

Tissue grafts were printed according to specified dimensions of 10 × 10 × 3 mm^3^, utilizing a 25% fill ratio in a hexagonal pattern. The BioX printer was operated under the following conditions: an extrusion pressure ranging from 30 to 50 kPa, a 20 G plastic conical tip, a cartridge thermostatted at 22 °C, a pre-flow of −300 ms, a head movement speed of 5 mm/s, and a bed temperature of 10 °C. Subsequent to bioprinting, each structure underwent photocuring using the BioX 405 photocuring module at 100% power, 4 cm height, for 60 s.

### 4.5. Tendon Cell Viability within Bioprinted Grafts

The viability of tendon cells encapsulated in PRP-bioink was evaluated using the CyQUANT TN XTT Cell Viability Assay (Invitrogen, Thermo Fisher, Waltham, MA, USA), following the guidelines provided by the manufacturer. The bioinks were combined with a concentrated tenocyte cell suspension in a 10:1 ratio, resulting in a final tenocyte concentration of 500,000 cells/mL. Following thorough mixing, 30 µL of the bioink mixture was added to each well of a 96-well flat-bottom, non-treated plate. Subsequently, the bioinks were photocrosslinked using the UV module of the BIOX printer for 20 s at 100% intensity and at a height of 4 cm to prevent cell deposition in the bottom of the wells.

Upon completion of photocuring, 70 µL of culture medium was added to each well. At the designated incubation time points, 70 µL of XTT working solution was placed into each well. After incubating for four hours in the cell culture incubator, absorbances at 660 and 450 nm were measured using a plate reader. The individual well’s specific absorbance was determined by subtracting Abs450Blank from Abs450Test and subsequently subtracting the obtained result from Abs660Test. Each experiment was conducted in triplicate.

### 4.6. In Vitro Evaluation of Graft Biology with and without Inflammation

GelMa grafts enriched with PRP and loaded with tenocytes were prepared to assess the impact of PRP augmentation within a non-inflamed or inflamed environment. Nude grafts or PRP grafts (GelMa or GelMA infused with PRP), both loaded with tenocytes (3.3 × 10^5^ cells/mL final concentration), were submerged in basal culture medium (DMEM:F12) with or without 50 ng/mL IL-1b. For control purposes, we used nude grafts and inflamed nude grafts. By doing so, we calculated the ratio of PRP graft/nude graft and the ratio of both grafts under inflammatory conditions. After 96 h of culturing at 37 °C with 5% CO_2_, the conditioned medium was harvested, centrifuged at 1000 rcf for 10 min, and the supernatants were preserved in cryotubes at −80 °C for protein microarrays, bioinformatics analyses, and ELISA confirmation.

### 4.7. Antibody-Based Protein Arrays

To evaluate the inflammatory cytokines and growth factors released by tenocytes within various hydrogel formulations and investigate the impact of inflammation, we utilized a multiplexing antibody array platform (126QAH-CAA-1000-1 RayBiotech Inc., Norcross, GA, USA). The array experiments were conducted following the manufacturer’s instructions, and the obtained results were quantified relative to positive controls. To address potential biological variability, plasma samples from six donors were pooled. The array scanning was carried out by the manufacturer’s service, and data analysis was performed using Quantibody Q-Analyzer Software version 8.40.4 (RayBiotech Peachtree Corners, Norcross, GA, USA)

### 4.8. Bioinformatic Analyses

#### 4.8.1. Search Tool for the Retrieval of Interacting Genes/Proteins (STRING) Database

Initially, we utilized the STRING database (version 11.5; string-db.org), a biological database and free web resource, to explore known protein interactions and enriched biological functions. Our selection criteria involved proteins over-expressed more than two-fold when comparing PRP grafts against nude grafts, representing associations with a high edge confidence of 0.9. The resulting figures depict robust networks, where edges signify both physical and functional protein associations. Disconnected nodes were omitted from the networks. Employing k-means methodology, the main network was clustered into four specified clusters. Enriched biological functions were illustrated against –LOG10 (FDR), with FDR representing the false discovery rate.

#### 4.8.2. Ingenuity Pathway Analysis (IPA^®^)

In this analysis, proteins released from PRP grafts, investigated in the arrays, were annotated and categorized based on their Gene Ontology (GO) functions. To assess the impact of PRP, PRP grafts, or inflamed PRP grafts, comparisons were made against nude grafts or inflamed nude grafts, respectively. Fold changes in protein expression were calculated in each case. 

The dataset from the multiplexing platform underwent analysis within the context of a comprehensive, structured collection of observations, incorporating nearly 5 million curated findings from various experimental settings in the biomedical literature or integrated from third-party databases. The Ingenuity Pathway Analysis Software QIAGEN IPA (QIAGEN Inc., https://digitalinsights.qiagen.com/IPA, accessed on 22 April 2024); Qiagen, Hilden, Germany) was employed for this analysis, utilizing algorithms to predict signal pathway networks and canonical pathways. The core analysis module identified activation or inhibition of canonical pathways, computed based on the Z-score algorithm of IPA^®^ and compared with an idealized activation or inhibition pattern for a signaling pathway or biological function. The comparison analysis module was utilized to compare different experimental conditions.

### 4.9. Angiogenic Assays

#### 4.9.1. Chemotaxis of HUVEC towards PRP Stimuli

Cells were seeded at a confluence of 3,000,000 cells/mL. Following the instructions of the Ibidi Chemotaxis Chamber, 6 µL was introduced into the central channel.

One hour after seeding the cells, both reservoirs, right (R) and left (L), were filled with PRP secretome versus DMEM (+/−) or DMEM vs. DMEM (−/−) or PRP secretome vs. PRP secretome (+/+) to test the effect of PRP on HUVEC chemotaxis. Images were then taken every 10 min for 24 h using the Zeiss Apotome with a 10× magnification objective. Ibidi software (https://ibidi.com/chemotaxis-analysis/171-chemotaxis-and-migration-tool.html, accessed on 22 April 2024) was used to calculate perpendicular and parallel Forward Migration Indexes (FMI) and the Rayleigh test.

#### 4.9.2. Matrigel Assays: HUVEC Exposed to PRP or Conditioned Media by Inflamed Tenocytes

Angiogenesis assays were performed in a 15-well-chambered coverslip (µ-Slide 15 Well 3D Ibidi 81506) according to the manufacturer’s protocol (Ibidi Co., Gräfelting, Germany). In each well, 10 µL of cold matrigel (growth factor-reduced—phenol-free; Corning 356231) was added and allowed to polymerize for at least 1 h in a cell culture incubator in a humid chamber. After polymerization, 20000 HUVEC were added to the matrigel bed, avoiding direct contact, in a volume no greater than 5 µL. Conditioned media from the cell-loaded grafts were quickly added to each well to a final volume of up to 50 µL and thoroughly pipetted up and down to achieve homogeneous cell distribution. HUVEC were allowed to attach to the matrigel in the culture incubator for 6 h. Cells were stained with CellMask™ Plasma Membrane Green Stain (Thermo Fisher C37608, Waltham, MA, USA) and counterstained with Hoechst 33342 (BioRad PureBlu™ Nuclear Staining Dye, Hercules, CA, USA). Live cell fluorescence images were captured in a Zeiss Axio Observer.Z1 Apotome 2.0, Axiocam 503M camera, Plan Neofluor 20×, 40×.

## Figures and Tables

**Table 1:** This table shows the most representative canonical pathways and the corresponding Z-score for PRP grafts in two contexts: non-inflammatory and inflamed environments.

	PRP Graft	Inflamed PRP Graft
HIF1α Signaling	4.082	3.266
Neuroinflammation Signaling Pathway	3.656	2.694
IL-33 Signaling Pathway	3.53	3.024
S100 Family Signaling Pathway	3.395	3.479
Osteoarthritis Pathway	3.272	2.502
IL-17 Signaling	3.244	3.647
IL-8 Signaling	3	2.84
IL-6 Signaling	2.524	2.982
TGF-β Signaling	2.449	1.633
Chemokine Signaling	2.333	3
Inhibition of Angiogenesis by TSP1	2	2.236
p38 MAPK Signaling	1.807	2.84
NF-kB Pathway	1.976	3.413
VEGF Family Ligand–Receptor Interactions	0.447	2.236
VEGF Signaling	0.816	2.449
CD40 Signaling	1	2
Toll-like Receptor Signaling	1.265	2.111
IL-1 Family Signaling	0.302	2.111
IL-10 Signaling	−2.4	−2.357

Identification of functional biological processes that are significantly activated/inhibited based on Z-score algorithm. Z-scores equal to or exceeding 2 indicate predictions of activation, whereas predictions of inhibition are assigned to z-scores equal to or less than −2.

## Data Availability

The data presented in this study are available on request from the corresponding author.

## Abbreviations

CM	conditioned media
ECM	extracellular matrix
FN	Fibronectin
GelMA	methacrylate gelatin
HGF	Hepatocyte Growth Factor
HIF-1a	Hypoxia Inducible Factor alpha
HUVEC	Human Umbilical Vein Endothelial Cells
ICAM	Intercellular Adhesion Molecule
IFNG	Interferon gamma
IGF-1	insulin-like growth factor
IL	Interleukin
IPA	Ingenuity Pathway Analyses
KDR	Kinase Domain Receptor
LAP	Lithium phenyl-2,4,6- Trimethylbenzoylphosphinate
MA	methacrylic anhydride
MAPK	mitogen-activated protein kinase
MCP-1	Monocyte Chemotactic Protein 1
MMP	Matrix Metalloproteinase
NF-kB	nuclear factor kappa light chain enhancer of activated B cells
PDGF	Platelet-Derived Growth Factor
PF4	Platelet Factor-4
PPI	protein–protein interactions
PPP	platelet-poor plasma
PRP	platelet-rich plasma
PTGS	Prostaglandin Endoperoxide Synthase
RANTES	Regulated Upon Activation, Normal T Cell Expressed And Presumably Secreted
SELL	Selectin
TGF-β	Transforming Growth Factor
TLR	Toll-like Receptors
TNF	Tumor Necrosis Factor
TRAF	TNF Receptor-Associated Factor
TSP-1	Thrombospondin
VCAM	Vascular Cell Adhesion Molecule
VEGF	Vascular Endothelial Growth Factor
